# “Motherese” Prosody in Fetal-Directed Speech: An Exploratory Study Using Automatic Social Signal Processing

**DOI:** 10.3389/fpsyg.2021.646170

**Published:** 2021-03-09

**Authors:** Erika Parlato-Oliveira, Catherine Saint-Georges, David Cohen, Hugues Pellerin, Isabella Marques Pereira, Catherine Fouillet, Mohamed Chetouani, Marc Dommergues, Sylvie Viaux-Savelon

**Affiliations:** ^1^School of Medicine, Universidade Federal de Minas Gerais, Belo Horizonte, Brazil; ^2^CRPMS, Université de Paris, Paris, France; ^3^Institut des Systèmes Intelligents et de Robotiques (ISIR), équipe Perception, Interaction, et Robotiques Sociales (PIRoS), Sorbonne Université, Paris, France; ^4^Service de Psychiatrie de l'Enfant et de l'Adolescent, APHP Pitié Salpêtrière, Sorbonne Université, Paris, France; ^5^Service de Gynécologie Obstétrique, APHP Pitié Salpêtrière, Sorbonne Université, Paris, France

**Keywords:** motherese, prenatal, mother-fetus interaction, fetal-directed speech, machine learning, social signal processing

## Abstract

**Introduction:** Motherese, or emotional infant directed speech (IDS), is the specific form of speech used by parents to address their infants. The prosody of IDS has affective properties, expresses caregiver involvement, is a marker of caregiver-infant interaction quality. IDS prosodic characteristics can be detected with automatic analysis. We aimed to explore whether pregnant women “speak” to their unborn baby, whether they use motherese while speaking and whether anxio-depressive or obstetrical status impacts speaking to the fetus.

**Participants and Methods:** We conducted an observational study of pregnant women with gestational ages from 26 to 38 weeks. Women were recruited in a university hospital department of obstetrics. Thirty-five women agreed to participate in the study, and 26 audio records were exploitable. We collected obstetrical and sociodemographic data, pregnancy outcomes, anxiety and depressive status using the Covy and Raskin Scales, and life events using the Sensations During Pregnancy and Life Event Questionnaire. Each participant was left alone with an audio recorder with a recommendation to feel free to speak to her fetus as she would have done at home. The recording was stopped after 3 min. Audio recordings were analyzed by two methods: psycholinguist experts' annotation and computational objective automatic analyses.

**Results:** Most mothers (89%) reported speaking to their fetuses. We found a correlation between maternal first perceptions of fetal movements and the start of mother's speaking to fetus. Motherese prosody was detected with both annotation and automatic analysis with a significant correlation between the two methods. In this exploratory study, motherese use was not associated with maternal anxiodepressive or obstetrical status. However, the more future mothers were depressed, the less they spoke with their fetuses during the recording.

**Conclusion:** Fetal directed speech (FDS) can be detected during pregnancy, and it contains a period of prosody that shares the same characteristics of motherese that can be described as prenatal motherese or emotional fetal-directed speech (e-FDS). This means that pregnant women start using motherese much earlier than expected. FDS seems to be correlated with maternal first perceptions of fetal movements and depression scores. However, more research is needed to confirm these exploratory results.

## Introduction

Infant-directed speech (IDS) or motherese is a specific register, which includes peculiar prosodic characteristics, that parents or caregivers often use when speaking to infants (Fernald and Simon, 1984; Fisher and Tokura, 1995; Spinelli et al., 2017). The use of motherese progressively increases as the baby grows and then usually decreases and disappears when the child becomes able to communicate verbally (Saint-Georges et al., 2013). IDS has been studied extensively across a number of interactive situations and contexts, especially by researchers interested in understanding language acquisition. IDS is also a marker of the parent infant interaction quality. Motherese characteristics have been shown to be linked with emotional prosody characteristics (Trainor et al., 2000). Behavioral studies have shown that infants prefer and respond better to motherese than to regular prosody, typical of adult directed speech (Fernald and Kuhl, 1987; Dupoux and Mehler, 1990; Saint-Georges et al., 2013; Outters et al., 2020). IDS has affective properties, expresses parental involvement, and contributes to regulating caregiver-infant interactions (Cohen et al., 2013). Thus, IDS is part of an interactive loop that may play an important role in infants' cognitive and social development (Saint-Georges et al., 2013). Experimental data suggest that very young infants in their first month of life (Cooper and Aslin, 1990; Cooper, 1993) or in their first week (Ramus, 1999) and even neonates (Saito et al., 2007) are sensitive to this prosody.

The *in utero* period has been less explored. A recent study (Bartha-Doering et al., 2019) suggested that neural discrimination of speech begins *in utero*. Some reports also show that future mothers speak to their fetus (DeCasper et al., 1994). In addition, parents observing their fetuses during ultrasound prenatal screening were shown to present mirroring movement activities (Ammaniti et al., 2010). These studies suggest that motherese may already be present in the prenatal period and may be associated with mother involvement and emotional tone regarding prenatal interactions. However, motherese has not been clearly demonstrated during the prenatal period.

With the development of automatized methods of social signal processing, machine-learning methods can now detect the acoustic characteristics of emotional speech, such as motherese, in the human voice. It can distinguish motherese sequences from adult-directed speech (Mahdhaoui et al., 2011). Traditionally, the design of computerized classifiers aims to capture supra-segmental features like pitch (fundamental frequency), duration, energy of vocalizations as well as global dynamics of spectrum (Williams and Stevens, 1972; Sherer, 1986; Chetouani et al., 2014). Mel frequency cepstral coefficients (MFCC) capture short-term dynamics of spectrum and are termed segmental features. The motherese detection algorithm system exploits the combination of two classifiers, segmental and supra-segmental, that are weighted and fused to reach best classification rates (Mahdhaoui et al., 2011). Previous works have shown that these methods can contribute to exploring parent-infant interactions in video/audio recordings in natural or experimental settings (Cohen et al., 2013; Weisman et al., 2016; Bourvis et al., 2018). Moreover, these automatic analyses of motherese have contributed to state the universality of the emotional prosodic characteristics of IDS across languages (Parlato et al., 2020).

Here, we describe an exploratory observational study based on interviews and audio recording of volunteer pregnant women with the following aims: (1.1) to determine whether pregnant women speak to their unborn baby, (1.2) if so, to determine, if they speak using motherese prosody or not, with two methods (1.3) with prosody analyzed by clinical experts, and (1.4) using computational analysis of speech with machine learning method. In addition, (2) we will assess if prenatal stress, obstetrical or fetal complications, and future mother emotional state would influence the quantity and characteristics of mother's prosody (Watson et al., 2002; Viaux-Savelon et al., 2012).

## Methods

### Participants and Ethics

From September 2013 to January 2014, we proposed to pregnant women attending the prenatal clinic of the Pitié-Salpêtrière University Hospital in Paris, France, to participate in a survey on maternal speech. They received oral and written information explaining that their participation would require filling out self-questionnaires, answering questions related to their emotional status, and being audiotaped when speaking to their future baby. The study was approved by the local Ethical Committee (CPPIDF6) under the number n°09012014. All participants gave a written consent. The inclusion criteria were mothers aged 18 or above, with a gestational age of 26–38 weeks, and able to understand the protocol. Indeed, during this period of pregnancy, future mothers are less concerned by the fetus viability. They begin preparing their meeting and relationship with the future child with more dreams and more detailed representations about the future baby (Ammaniti et al., 2000). Provided women were fluent in French, they could be included even if French was not their native language. Exclusion criteria were mental disorders and absence of health coverage according to the French ethical rules that require that studies be carried out only on people with health coverage. The mental disorder information were extracted from the obstetric record. Mental disorders were considered to be present if the pregnant woman was cared or treated for mental disorder by specialist before the pregnancy.

### Data Collection

#### Clinical Data Collection

We collected several clinical variables. Social and demographic characteristics included age, parity, marital status, native language, education level, and occupation. We also assessed life events using the Sensations During Pregnancy and Life Event Questionnaire (Tordjman et al., 2004). Fetal-oriented interaction variables included gestational age when the pregnant women reported spontaneous moment of speaking to their infant to come and the gestational age they started perceiving fetal movement. To assess maternal anxiety-depression status, we used two specific scales. Maternal anxiety was assessed using the Covi anxiety scale, a questionnaire completed by the investigator (EP), based on clinical assessment. This score ranges from 0 (no anxiety) to 12 (high anxiety), with a threshold of six defining clinically relevant anxiety (Covi, 1986). Depression was assessed using the RASKIN score based on clinical assessment. This score ranges from 0 (no depression) to 12 (high depression), with a threshold of six defining clinically relevant depression (Raskin and Crook, 1976). In case of clinically relevant depression or anxiety, we planned that the investigator would warn the doctor or midwife in charge of the patient to organize adequate follow-up.

Finally, we retrospectively recorded medical history and obstetrical outcomes after birth based on obstetrical and neonatal records by professionals who were blinded to the audio analysis. Variables are listed in Table 1 (pregnancy and delivery sections). Breastfeeding initiation was collected as some studies have pointed out that stress events during pregnancy may influence breastfeeding initiation or duration (Evers et al., 1998; Figueiredo et al., 2013).

**Table 1:** Description of study participants *N* = 35.

***Sociodemographics***	
Age: mean (SD) [range] in years	32.34 (6.4) [18.9–42.66]
Marital status: single/in couple	7 (20%)/28 (80%)
Working status: No/Working/Student	6 (17.1%)/27 (77.1%)/2 (5.7%)
Years of education: ≤12/>12 years	8 (22.9%)/27 (77.1%)
Mother tongue: French/other	30 (85.7%)/5 (14.3%)
***Life events***	
Number of life events: mean (SD) [range]	7.69 (5.95) [0–24]
Significant obstetrical history: n (%)	21 (60%)
Significant medical history: n (%)	23 (65.7%)
***Pregnancy***	
Gestational age at study recruitment: mean (SD) [range] in weeks	32.45 (3.69) [22–38]
At least one fetal risk: n (%)	17 (49%)
Ultrasound soft marker: n (%)	2 (6%)
At least one maternal risk: n (%)	21 (60%)
Fetus gender: Female/Male	22 (63%)/13 (37%)
Complication during pregnancy: n (%)	13 (37%)
Global risk: No/Fetus only/Mother only/Both	7 (20%)/4 (11.4%)/11 (31.4%)/13 (37.1%)
***Psychopathology***	
Covy anxiety score: mean (SD) [range]	1.7 (2.15) [0–6]
Raskin depression score: mean (SD) [range]	1.67 (1.84) [0–6]
***Fetal oriented interaction variables***	
Mother gestational age when first sentences were addressed to the fetus: mean (SD) [range] in months	3.63 (1.64) [0–6]
Mother declare spontaneously speaking to fetus: never or rarely/frequently/missing data	9 (25.7%)/22 (62.9%)/4 (11.4%)
Mother gestational age when they first perceived their fetus moving: mean (SD) [range] in months	2.83 (1.89) [1–7]
***Delivery***	
Gestation duration: mean (SD) [range] in weeks	38.82 (2.23) [29.1–41.5]
Baby weight at birth: mean (SD) [range] in g	3047.6 (684.32) [2140–3850]
5 min APGAR: mean (SD) [range]	9.91 (0.37) [8–10]
Breast feeding: n (%)	29 (83%)
Mode of delivery: Basse/VBI/Anticipated Caesarian/Emergency Caesarian/Missing data	17 (48.6%)/5 (14.3%)/9 (25.7%)/3 (8.6%)/1 (2.9%)

#### Audio Data Collection and Analyses

After the questionnaires were completed, the investigator invited the participant to sit in a quiet room, independent of the prenatal clinic suite. The participant was left alone with a recorder (Zoom recorder AT170 PRO-Sony) lying on a nearby table. She was asked to feel free to speak or not to her baby, as she would have done at home. The mother was taken to a quiet room with a comfortable chair and invited to speak with her fetus, if only she wanted to. The interviewer would turn on the recorder and leave the room, so as not to intimidate the mother and allow the environment to be as close as possible to the mother's usual situation with her fetus. The recording was stopped after 3 min. Audio recordings were analyzed at two levels: (i) maternal vocalization characteristics (low-level features) and (ii) affective speech analysis (high-level audio features).

#### Maternal Vocalization Characteristic

During a dialogue or a monolog, vocalization can be characterized by a series of quantitative parameters that allow describing the features and their dynamics. For our survey, we adapted this method to the monolog uttered by the mother, making the hypothesis that what we recorded was the equivalent of a dialogue between the mother and her unborn child. We first segmented and annotated the mothers' vocalization based on the Weisman et al. (2016) method. Two experts (EP and IR), one linguist and one speech therapist, listened to every 3-min recording. Using the ELAN EUDICO Linguistic Annotator (Institut Max-Planck, Nimègue, Nederland), they worked together to split them into segments of vocalization defined as continuous streams of speech with <150 ms of silence. Then, they labeled each segment as vocalization, laughing, singing, crying or other sounds. The maternal vocalizations consisted in the mother's recorded sound production directed or not to the fetus. For examples: “I'm very tired,” “I can't wait to see you,” “My baby, your room is ready, we await you with love,” “I am afraid about childbirth.” Maternal vocalization, maternal pause, and silence were extracted using an automated algorithm (for details see Bourvis et al., 2018). It calculated the duration of each segment and the amount of pause time within each 3-min recording, corresponding to the sum of silences >150 ms between two segments. Thus, we obtained the *maternal vocalization mean duration*, the *vocalization number during the 3-min window*, the *maternal pause mean duration* and the *vocalization ratio of time during the 3 min*.

#### Affective Speech Analysis

Each speech segment labeled “vocalization” by the experts was extracted as a digital audio sample, stored and submitted to affective speech analysis based on high-level audio features. The goal of this analysis was to categorize each vocalization as “motherese,” based on the presence of the emotional component of IDS, vs. “non-motherese,” which refers to prosody more typical of adult directed speech. This was achieved by two methods: expert evaluation by listening to the audio samples of the segments labeled “vocalization” and computational automatic assessment of the same digital samples.

For the manual qualitative annotation, the two experts (EPO and IMP) worked independently to assess the presence of motherese characteristics in each vocalization segment. Interrater agreement between the two independent raters was calculated on the whole sample of vocalizations and was found to equal 80%. In case of disagreement, they listened again together to the remaining segments with no agreement (20%) and reached a consensus. This method allowed us to obtain a unique manual label for each vocalization segment.

Automated labeling for motherese or non-motherese was performed using an *ad hoc* algorithm developed in the ISIR *(Institut des Systèmes Intelligents et de Robotiques)* laboratory in Paris. This motherese classifier, based on machine learning methods, uses both segmental (mel-frequency cepstrum coefficients, MFCCs) and suprasegmental (e.g., statistics with regard to fundamental frequency, energy, and duration) acoustic characteristics of speech and SVM (support vector machine) classifiers. The algorithm classifier was trained on a data set of both motherese and non-motherese. It can distinguish emotional sequences of motherese from normal speech (Mahdhaoui et al., 2011). In previous studies, it was able to identify motherese during early interaction in both experimental (Weisman et al., 2016; Bourvis et al., 2018) and natural settings (Cohen et al., 2013), in both mothers and fathers (Cohen et al., 2013; Weisman et al., 2016; Parlato et al., 2020), in various languages (Parlato et al., 2020), and in parents speaking to infants with later psychopathology (e.g., autism, Cohen et al., 2013).

Both motherese detection methods created two subclass labels of maternal vocalization: “motherese” labeled Emotional Fetal-Directed Speech (e-FDS) vs. “non-motherese” (non-e-FDS). Three variables were derived: e-FDS ratio during the 3 min, non-e-FDS ratio during the 3 min, and *e-FDS/vocalization ratio* (duration of “motherese” vocalization/duration of maternal vocalization).

### Statistical Analysis

Statistical analyses were performed using R Software, Version 2.12.2. For all tests, the level of significance alpha was fixed at 5%. Given the sample size and the exploratory nature of the study, we used univariate analysis only. Quantitative variables were presented as the mean, standard deviation, and range. Qualitative variables were presented as frequencies.

First, we explored the correlation between maternal first perception of fetal movements and first vocalizations to the fetus.

We then successively estimated the relationship between:

- Variable “*Vocalization ratio of time during the 3 min”* and the following variables: “depression score” (score Raskin), “anxiety score” (score Covy), “life events” and “fetal risk”.
- *Variable ratio Emotional-Fetal Directed Speech (e-FDS)/vocalization according to psycholinguist expert* and the following variables: “depression score” (score Raskin), “anxiety score” (score Covy), “life event” and “fetal risk”.

The relationship between two continuous variables was either tested using Pearson r or Spearman rho, depending on the validity of the assumptions. The relationship between a continuous and a binary variable (fetal risk) was either tested using the Welch *t*-test or Wilcoxon rank sum test, depending on the validity of the assumptions. Finally, we estimated the agreement between our experts' measures and the algorithm's measures using ICC (single random raters, ICC2) and calculated the 95% confidence interval (R psych package).

## Results

### Flow Chart

One hundred forty-five pregnant women were considered eligible from September 2013 to January 2014. Thirty-five of them agreed to participate in the study and were enrolled. Four women participated in the clinical part of the study but did not record audio data; five audio records were not exploitable because of technical difficulties. Thus, 26 audio records were used for analysis. The most frequent reason declared by the invited mothers to decline the participation to the research was the lack of available time, considering that the interview needed at least 1 h and 30 min. Indeed in order to avoid displacements, we proposed the study to mothers who were already present at the Hospital for a pregnancy consultation.

### Description of the Sample (*N* = 35)

Table 1 summarizes the study sample in terms of socio-demographics, life events and pregnancy risk factors, delivery, psychopathology and fetal-oriented interaction variables. Mothers presented a high number of life events and significant obstetrical (60%) and medical (65.7%) history: endocrinologic conditions (*N* = 7), multisystemic pathologies (*N* = 3), neurologic disorders (*N* = 4), psychiatric disorders (*N* = 2), uterine anomaly (*N* = 2), social precariousness (*N* = 2), and cardiac anomaly (*N* = 1). Regarding fetal risk, we found trisomy 21 risk (*N* = 3), intrauterine growth retardation (*N* = 2), premature delivery threats (*N* = 2), cardiovascular anomalies (*N* = 1), drug exposure (*N* = 1), and prior history of neonatal death (*N* = 1). This could be related to recruitment inside a free public university hospital in a maternity unit specializing in complex cases.

Nevertheless, our sample presented a low mean level of anxiety and depression scores and a high percentage of breastfeeding compared to the French general population (68.1–70.5% in the general population with 59% exclusive breastfeeding https://drees.solidarites-sante.gouv.fr/IMG/pdf/dt68-sources_et_methodes.pdf; Kersuzan et al., 2014).

### Analysis of Fetal-Oriented Interaction Variables (*N* = 35)

Mothers declared to start speaking or vocalizing to their fetus on average at 3.63 (±1.64) months during pregnancy. Additionally, they started perceiving fetal movement on average at 2.83 (±1.89) months during pregnancy. We found a significant correlation between speaking to the fetus and perceiving fetal movements (**Figure 2**, right).

**Figure 1:**
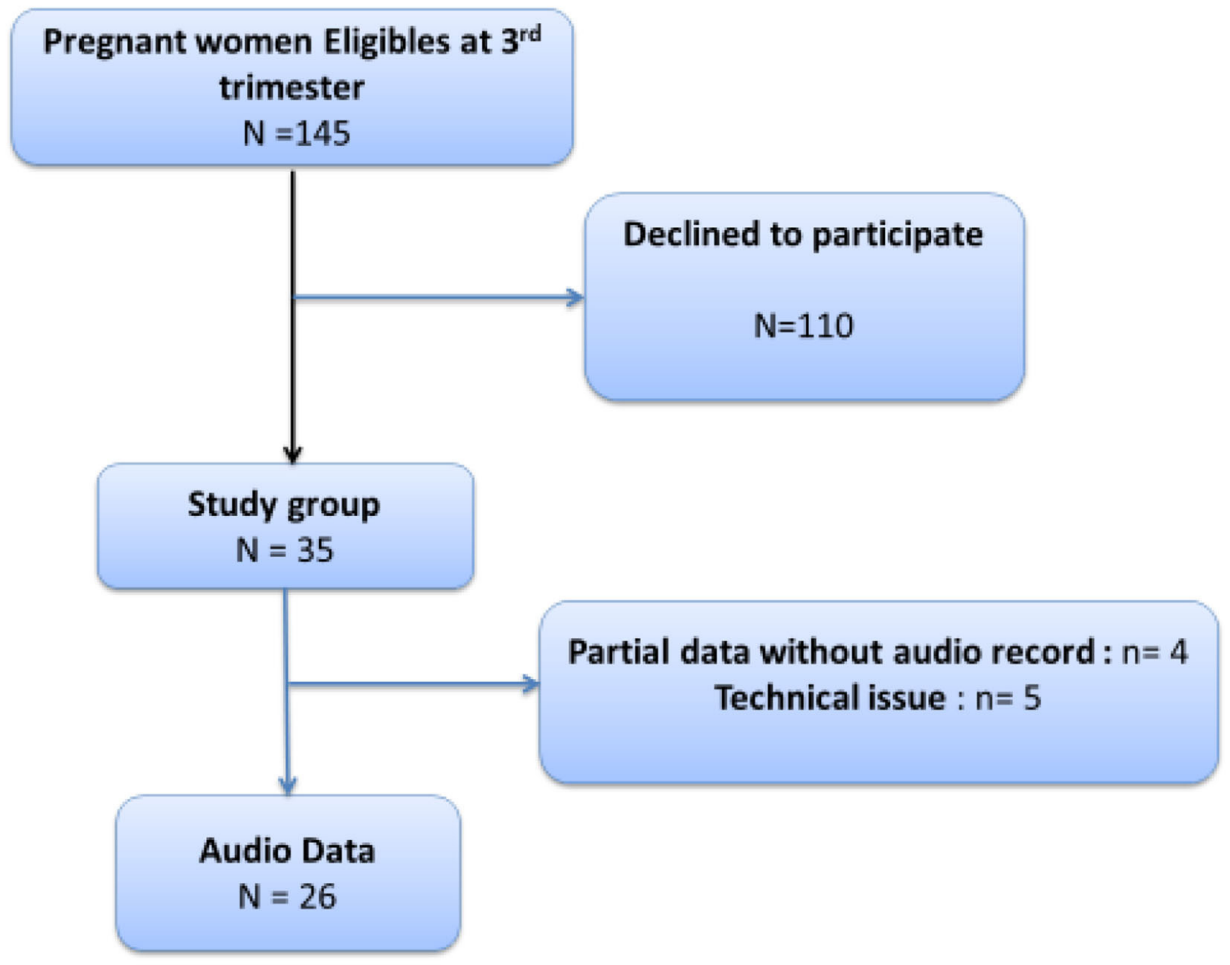
Flow chart of recruitment.

Regarding audio analyses, only 26 mothers were included because of technical issues (see Figure 1 flow chart). For low-level audio analysis (quantitative speech analysis), a total of 856 vocalization segments (mean vocalization number = 32.92) were detected. The duration of the vocalizations ranged from 0 to 3.95 s during the 3-min audio record (Table 2). The vocalization ratio (vocalization time during the 3 min) ranged from 0 to 65%. Indeed, two mothers did not speak during the 3-min recording.

**Table 2:** Maternal vocalization characteristics during the experiment (*N* = 26).

Vocalization mean duration: mean (SD) [range]	1.89 (0.92) [0–3.95]
Vocalization number during the 3 min window: mean (SD) [range]	32.92 (16.66) [0–59]
Maternal Pause mean duration: mean (SD) [range]	4.69 (6.3) [0–28.38]
Vocalization ratio of time during the 3 min: mean (SD) [range]	0.35 (0.19) [0–0.65]
***Emotional-Fetal Directed Speech according to automatic classification***	
e-FDS ratio during the 3 min: mean (SD) [range]	0.12 (0.17) [0–0.65]
Non e-FDS ratio during the 3 min: mean (SD) [range]	0.23 (0.18) [0–0.64]
e-FDS/vocalization ratio: mean (SD) [range]	0.29 (0.3) [0–1]
***Emotional-Fetal Directed Speech according to psycholinguist expert***	
e-FDS ratio during the 3 min: mean (SD) [range]	0.14 (0.16) [0–0.51]
Non e-FDS ratio during the 3 min: mean (SD) [range]	0.21 (0.16) [0–0.62]
e-FDS/vocalization ratio: mean (SD) [range]	0.34 (0.34) [0–1]

**Figure 2:**
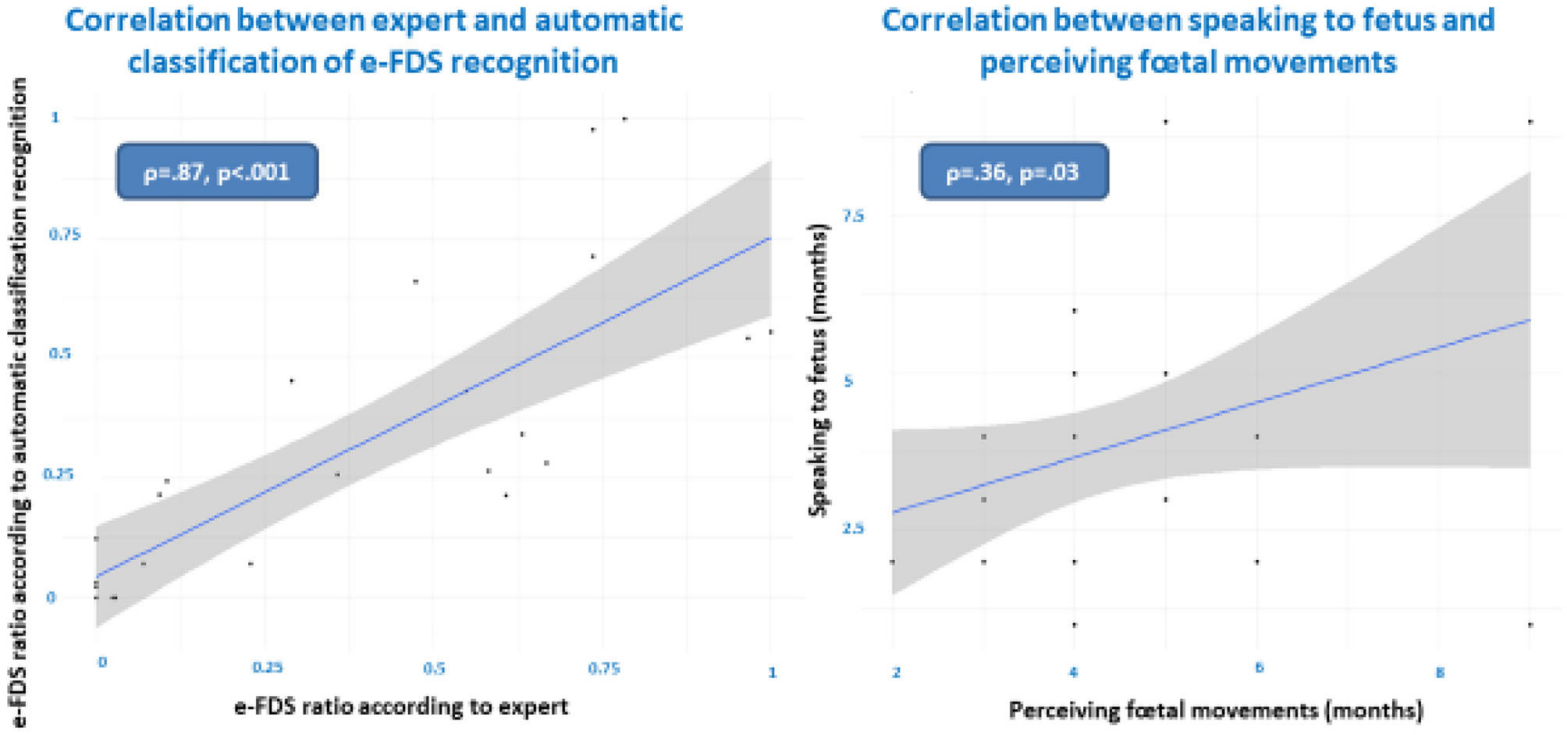
Correlations between expert and automatic classification of emotional fetal directed speech (e-FDS, left) and between speaking to fetus and perceiving fetal movements (right).

Regarding high-level audio analysis (qualitative affective speech analysis), two complementary methods were performed: a qualitative manual annotation of maternal vocalization to the fetus by two expert psycholinguists and an automatic classification. In both clinical expert and automatized classifications, we found that pregnant women when speaking to their fetus (FDS) used sometimes a specific prosody that usually characterized motherese (or emotional IDS), which we called emotional fetal directed speech (e-FDS). We called the FDS without motherese characteristics “non-e-FDS.” The automatic classification yielded a mean e-FDS ratio during the 3 min of 0.12, whereas the expert classification found a mean e-FDS ratio during the 3 min of 0.14 (Table 2). Figure 2 (left) shows the strong and significant correlation between expert and automatic classification on e-FDS recognition. We also calculated the intraclass correlation (ICC) between the “two” raters (the expert and the algorithm) and found a good and very significant ICC (ICC = 0.79 (95% CI: 0.59–0.90), *p* < 0.001).

### Correlation of Maternal Audio Data With Maternofetal Characteristics and Anxiety Depression Status (*N* = 26)

Given the limited sample size, we used only exploratory univariate analysis to address whether some stress or psychopathological variables could influence the ability to produce e-FDS. We found no association between speaking to the fetus (whether prosody had characteristics of e-FDS or not) and being a fetus at risk during pregnancy (correlation ratio = 0.36 (±0.2) and 0.33 (±0.2), respectively, *t*-test, *p* = 0.69).

**Figure 3:**
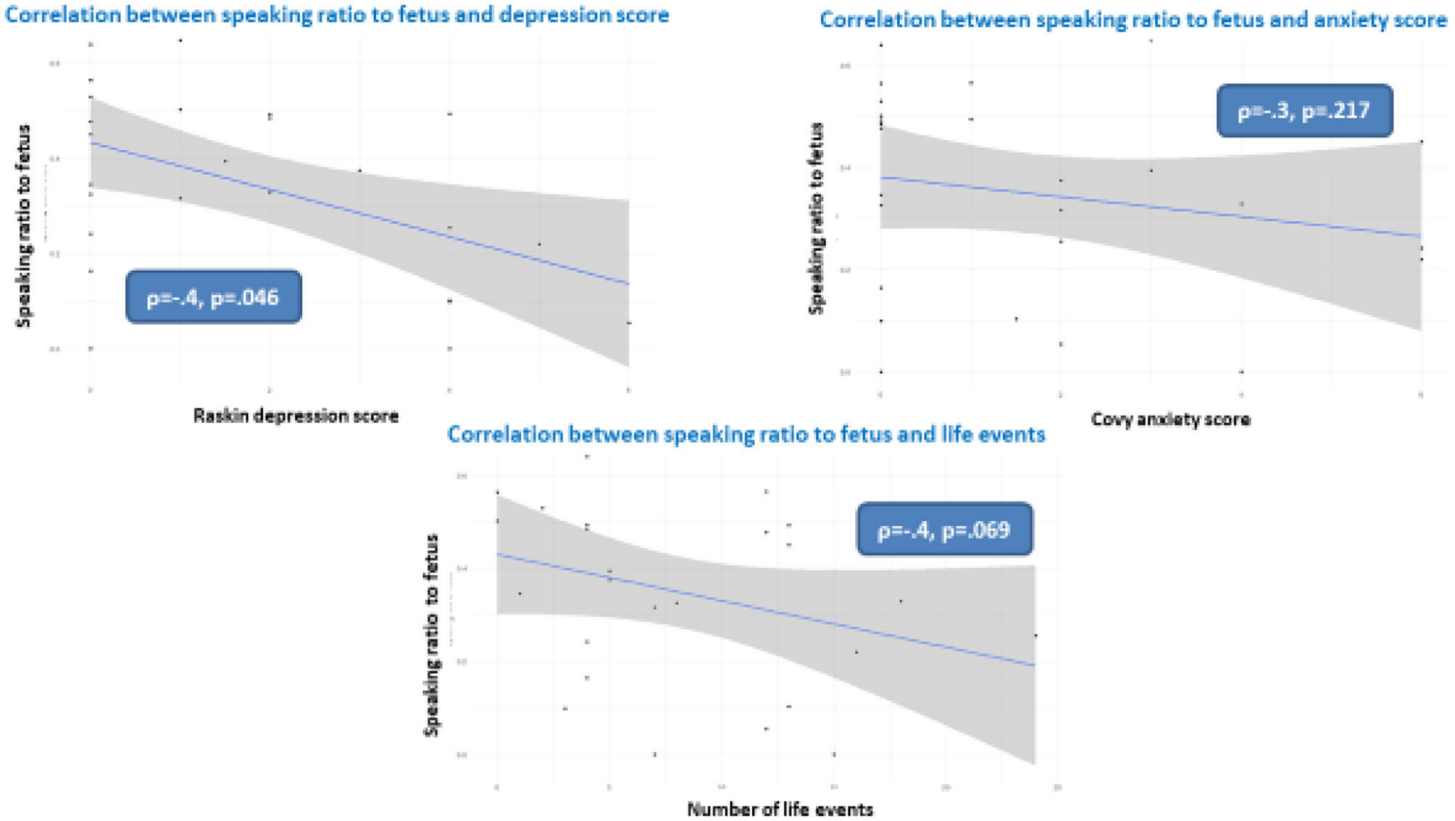
Correlations between speaking to fetus and Raskin depression score (upper left), Covy anxiety ratio (upper right) and number of mother stressful life events.

Figure 3 shows the correlation between the speaking-to-fetus ratio and the Covy anxiety score, Raskin depression score and number of maternal stressful events. As shown, we found no correlation with the Covy anxiety score, a tendential negative correlation with the number of maternal stressful events, and a significant negative correlation with the Raskin depression score (ρ = −0.4, *p* = 0.046), meaning that the more the future mothers were depressed during pregnancy, the less they spoke to their fetuses during the experiment.

We performed the same analyses using only the e-FDS/vocalization ratio. None of the variables modulated the e-FDS/vocalization ratio, meaning that the impact of the Raskin depression score was on speaking to the fetus as a whole whether the pregnant women had e-FDS prosody or not. However, there was a trend toward a negative correlation between the e-FDS/vocalization ratio and the number of maternal stressful events (rho = −0.421, *p* = 0.072).

## Discussion

### Can We Define Emotional Fetal Directed Speech (e-FDS)?

To answer this question, we proposed to address two different issues: (1) Is the mother speaking to the fetus during the experiment truly oriented toward the fetus? And (2) does FDS include some sequences that share the same prosodic characteristics of postnatal IDS (motherese)?

To address this first question (Is the mother speaking to the fetus during the experiment truly oriented toward the fetus?), we explored whether the pregnant women reported spontaneous moments of speaking and vocalization with their infant to come. For the mothers who reported doing so (*n* = 26), mothers started speaking or vocalizing with their fetus on average at 3.63 months during pregnancy (Table 1). Additionally, they started perceiving fetal movement on average at 2.83 months during pregnancy. This means that they could feel physically the existence of their fetus before they reported speaking to their fetus. Given the significant correlation between speaking to the fetus and perceiving fetal movement gestational ages (Figure 2, right), we can hypothesize that speaking to the fetus was indeed oriented toward the fetus. This result supports the hypothesis of a preliminary dialogue between future mothers and their fetus, as shown when mothers observed fetal movements during ultrasound scans that were interpreted by mothers as a response or solicitation from the fetus. Mirroring movements were seen as motor turn taking (Ammaniti et al., 2010). We believe that the current results on e-FDS are in the same vein and support the idea that prenatal development influences maternal infant attachment (Ammaniti et al., 2014; Feldman, 2016; Malm et al., 2016) and maternal representations of her future child (Viaux-Savelon et al., 2012, 2020).

Regarding the second question (do pregnant mothers sometimes use a motherese prosody (or here e-FDS) when speaking to their fetus?), our results show that futures mothers can use motherese prosody in their fetal-directed speech. The “manual” study of acoustic components of the voice takes a very long time and only allows the study of very short voice segments. The use of an automatic classifier allows extensive study of all vocalizations based on their acoustic characteristics and open perspectives for larger studies, and the machine learning classifier remains blind to the experiment or context. In this study, in addition to the expert “manual” categorization, the presence of e-FDS is confirmed by automatic measures that are strongly objective. Indeed, we found a strong and significant correlation between expert and automatic classification on e-FDS recognition (ρ = 0.87 *p* < 0.01) and a good and very significant ICC between expert and algorithm [ICC = 0.79 (95% CI: 0.59–0.90), *p* < 0.001]. This methodology of motherese detection using an algorithm has already shown robustness, as we have been able to distinguish motherese in early interaction with children with pathological outcome (Cohen et al., 2013), with both mothers and fathers (Weisman et al., 2016), and in five different languages (Parlato et al., 2020). Here, automatic annotation was useful to confirm that the prosody used during FDS shared the same characteristics of motherese. Manual and automatic labeling comparison realizes a validation of the two methods.

### Does Maternal Anxiety Depression Status Influence the Quality and Quantity of Fetal Directed Speech?

As expected, despite the limited sample size, the results show that the more the future mothers were depressed during pregnancy, the less they spoke to their fetuses during the experiment (Figure 3). We also found a tendency for a significant negative correlation between stressful life events and the speaking-to-fetus ratio (*p* = 0.69). These results are contingent with previous studies that have shown the impact of maternal prenatal states. Prenatal stress, particularly concerning prenatal diagnosis, increases the level of anxiety, disrupts the emotional investment of the parents toward the fetus (Watson et al., 2002; Petersen and Jahn, 2008; Kaasen et al., 2010) and disrupts parent-infant interactions after birth (Viaux-Savelon et al., 2012). In addition, pregnant women with depressive and anxiety symptoms talk and sing less to their fetuses (Hernandez-Reif et al., 2018).

Regarding fetal-directed speech *quality* (e-FDS or fetus-directed motherese), we found only a trend toward a negative correlation between the e-FDS/speaking to fetus ratio and the number of maternal stressful events. Thus, a high number of stressful events may reduce mothers' affective involvement with their future infant. We know that depressed mothers of young infants are not only less likely to speak with them (Herrera et al., 2004) but also more likely to display a reduced prosody of motherese with them (Bettes, 1988; Kaplan et al., 2001). Moreover, even when depressed mothers produce motherese, their infants fail to learn in response to their own-mother infant directed speech, despite normal competence (Kaplan et al., 1999, 2002). In our study with fetuses, we found that depressed mothers speak less to their fetus, but we did not find a correlation between depression score and e-FDS ratio. However, we cannot exclude that motherese quality could be poorer and less able to prepare language acquisition. As suggested in a recent study (Bartha-Doering et al., 2019), neural discrimination of speech could begin *in utero*. So we could expect that depression during the end of pregnancy may have repercussions on the first steps of language acquisition. However, given the small size of our sample and the exploratory nature of the study, we cannot conclude, and further studies with larger samples would be helpful.

Anxiety and depression status and a high level of stressful life events influence at least the quantity of fetal directed speech. Therefore, the quantity of fetal directed speech may be a sign to consider when detecting depression during pregnancy. Indeed, supporting these mothers in their investment toward the fetus and the future infant is compulsory for the prevention of later psychopathology (Mazzeschi et al., 2015; Røhder et al., 2020).

Finally, we found no significant association between speaking to the fetus (whether prosody had characteristics of e-FDS or not) and having a fetus at risk during pregnancy. This was not our hypothesis. However, the mothers' and fetuses' medical and obstetrical history of our population is very heterogeneous in this small sample size, and all risks may not be similar. In addition, the gestational age of the stressful event could also influence the impact on maternal representations and involvement. Again, a larger study would be necessary to better explore these factors with a comparison group according to the type of stress factor (e.g., mother complication/fetal complication/others) (Viaux-Savelon et al., 2012, 2020; Pisoni et al., 2016; Cuijlits et al., 2019).

### Study Limitations

As noted above, our sample was scarce (*N* = 26) and did not permit us to draw conclusions regarding the effects of various complex factors, such as maternal anxiety depression status or fetus risk. As many women declined participation in the study, we must discuss whether future mothers who agreed to participate could be more susceptible to speaking to their fetus than future mothers who declined participation. This study is only exploratory and used an experimental context. We also need to confirm that speaking to fetus also occurs spontaneously in more ecological contexts (e.g., at home). This might be achievable with automatic recording using portable devices for example. Additionally, we did not perform multivariate models to explore how relevant variables are robustly correlated or not. Nevertheless, it is important to note that one mother who declared before the audio recording she did not usually speak to her fetus actually spoke a lot to her during the recording. This may suggest that speaking to her fetus may be a widespread phenomenon.

## Conclusion

Fetal directed speech (FDS) can be detected during pregnancy, and it contains a period of prosody that shares the same characteristics of motherese that can be described as prenatal motherese or emotional fetus-directed speech (e-FDS). This means that pregnant women start using motherese much earlier than expected. FDS seems to be correlated with maternal first perceptions of fetal movements and depression scores. Although this study was exploratory, our results show that the more future mothers were depressed, the less they spoke to their fetuses during pregnancy. Therefore, the quantity of fetal directed speech may represent a useful sign for clinicians to detect prenatal depression and maternal involvement during pregnancy. However, more research (e.g., larger sample; prospective design with several timeline measures during pregnancy) is needed to confirm these exploratory results. Automatic audio detection and social signal processing should enable larger studies that explore prenatal emotional involvement with future infants.

## Data Availability Statement

The raw data supporting the conclusions of this article will be made available by the authors, without undue reservation.

## Ethics Statement

The studies involving human participants were reviewed and approved by the local Ethical Committee (CPPIDF6) under the number n°09012014. The patients/participants provided their written informed consent to participate in this study.

## Author Contributions

DC, SV-S, MD, and MC designed the study. CF, MD, EP-O, IP, and SV-S recruited the participants and assessed both obstetrical and psychological data. EP-O, SV-S, and CF performed the experiments. EP-O and IP assessed motherese prosody. CS-G, DC, and MC performed the automatic signal processing. DC and HP performed the statistical analysis. EP-O, DC, MD, and SV-S wrote the first draft of the manuscript. All authors contributed to the final version of the manuscript.

## Conflict of Interest

The authors declare that the research was conducted in the absence of any commercial or financial relationships that could be construed as a potential conflict of interest.
